# Defining and measuring acceptability of surgical interventions: A scoping review

**DOI:** 10.1371/journal.pone.0323738

**Published:** 2025-06-03

**Authors:** Sophie James, Jennie Lister, Joy Adamson, Catherine Hewitt, Antonina Yakimova, Andrew Mott, Helen Fulbright, Catriona McDaid

**Affiliations:** 1 York Trials Unit, Department of Health Sciences, University of York, York, United Kingdom; 2 Centre for Reviews and Dissemination, University of York, York, United Kingdom; IRCCS Burlo Garofolo Trieste, ITALY

## Abstract

**Background:**

Acceptability, in the context of healthcare interventions is a frequently used term, including in evaluations of surgical interventions. This reflects the importance of the concept to all stakeholders and significance to designing, implementing and evaluating interventions. Despite this, definitions and measurement of acceptability are not standardised, and acceptability is often poorly conceptualised. The aim of this scoping review was to identify how studies define, measure and report the acceptability of a surgical intervention.

**Methods:**

A scoping review was conducted adhering to the Joanna Briggs Institute guidelines and reported according to the Preferred Reporting Items for Systematic Reviews and Meta-Analyses extension for scoping reviews. A comprehensive search of MEDLINE; Embase:APA PsycInfo; EBHealth-KSR Evidence; Cochrane Central Register of Controlled Trials; International HTA database; ClinicalTrials.gov and WHO International Clinical Trials Registry Platform was conducted for the period January 2000 to November 2023. No language limits were applied.

**Results:**

Sixty-seven studies from 25 countries were included. The majority of studies (n = 60; 90%) did not provide a definition of acceptability. Various methods were used to collect data on acceptability, most frequently a questionnaire (n = 36; 54%), followed by qualitative interviews (n = 16; 24%). Thirty-three studies (49%) reported acceptability of the surgical intervention received to patients, nine (13%) reported hypothetical acceptability of the surgical intervention to patients, four (6%) reported acceptability to both patients and surgeons, and four studies (6%) the acceptability to surgeons alone.

**Conclusion:**

Studies assessing acceptability of a surgical intervention tended not to provide a definition of acceptability and demonstrated a lack of clarity in the use of acceptability in the context of surgical interventions. There was substantial variability in how and when acceptability was measured and from which perspective.

Further research is required to explore the most appropriate approaches to address variability and promote a more consistent conceptualisation and accurate measurement of acceptability in evaluations of surgical interventions.

## 1. Introduction

The acceptability of healthcare interventions is recognised as important across all stakeholders [1–3]. In the UK context, the acceptability of an intervention is often a requested outcome in National Institute of Health Research (NIHR) commissioning briefs. There are a number of definitions of acceptability in healthcare [3–5] which tend to be broad and nonspecific. A recent definition, developed alongside the Theoretical Framework of Acceptability (TFA) describes acceptability as:

“a multi-faceted construct that reflects the extent to which the people who provide or receive an intervention consider it to be appropriate, based on anticipated or experienced cognitive and emotional responses to the intervention” [6].

Several different ways of capturing acceptability have been explored, including through qualitative interviews and focus groups, quantitative patient reported outcome measures, or by proxy through the reporting of participants’ behaviours (e.g., giving consent) [5–9]. However, there is no agreed definition of acceptability and the concept is generally considered as vague [10], and not well measured [6,11].

Surgical interventions are a common, yet distinctive, form of healthcare intervention. There are more than six million surgical procedures taking place within the NHS annually [12] and over 300 million worldwide [13]. The acceptability of a surgical intervention to patients, or lack of acceptability, could affect their willingness to undergo a surgical procedure. Additionally, the IDEAL Framework [14] provides a structured evaluation pathway for surgical innovation, and recommends that evaluation and assessment of a surgery should include standards for acceptable delivery.

There is currently no conceptualisation of acceptability specific to surgery. There are existing definitions and frameworks developed for evaluating the acceptability of complex behavioural healthcare interventions, such as the TFA, [6] however, it is unclear whether these can be applied to surgical interventions [15]. Surgery has many different concomitant factors that may have implications for acceptability, for example variations in the skills of those delivering the care, the surgical wider team and setting, all of which can occur pre-, peri- or post-operatively [16,17].

There is uncertainty as to how best to assess the acceptability of surgical interventions. Acceptability is often grouped with closely related concepts such as patient satisfaction or treatment preference [1] and there remains a need for a better understanding of acceptability in this context. Therefore, we sought to answer the research question: how do studies evaluating the acceptability of a surgical intervention define, measure and report this? In order to achieve this and broadly map the existing evidence to identify, characterise and summarise this topic, rather than addressing feasibility, appropriateness or effectiveness, a scoping review was considered the most appropriate method [18].

### 1.1 Aim and objectives

The aim of this scoping review was to identify how studies define, measure and report acceptability of a surgical intervention. The specific objectives were:

1. To describe the definitions of acceptability used in these studies.
2. To describe the methods of collecting data on acceptability used in studies (qualitative and quantitative) and key contextual factors (e.g. who reported the acceptability, the stage of development of the intervention and when the measurement of acceptability had taken place).
3. To identify knowledge gaps in the literature on acceptability measurement for surgical interventions.

## 2. Methods

### 2.1 Protocol and registration

A scoping review was conducted according to best practice guidance from the Joanna Briggs Institute (JBI) Scoping Review Methodology Group [19]. The Preferred Reporting Items for Systematic Reviews and Meta-Analyses extension for scoping reviews (PRISMA-ScR) guidelines [20] were utilised in the reporting of this study (S1 Table). The protocol was published through the Open Science Framework (https://osf.io/uaxvp).

### 2.2 Eligibility criteria

The eligibility criteria are detailed in Table 1. Studies which reported measuring the acceptability of at least one surgical intervention were eligible for inclusion. Studies that approached acceptability using only proxy terms such as ‘feasibility’ alone were excluded [6]. Only studies published since 2000 were included to reflect contemporary practice for evaluation of surgical interventions.

**Table 1:** Eligibility criteria for the scoping review.

Inclusion criteria
• Full publication of results or protocol of a study which reports to measure or assess individuals’ views on acceptability of a surgical intervention. Using the definition of surgical intervention “those that cut or physically alter a patient’s tissues (whether using a scalpel, stapler, laser or another instrument or device) and involve the use of a sterile environment, anaesthesia, antiseptic conditions and suturing or stapling” [17].
• English language studies or able to be translated sufficiently for data extraction using Google translate [21].
• All study designs were included (observational, experimental/quasi-experimental, and qualitative designs)
• Published in or after the year 2000.
**Exclusion criteria**
• Studies that use the term acceptable to describe another outcome measure for example ‘acceptable toxicity levels,’ ‘acceptable adverse events,’ ‘acceptable morbidity and mortality’ or that report a global, general use of the word acceptable to describe outcomes for example ‘acceptable surgical outcomes,’ ‘acceptable treatment alternative’ or ‘acceptable clinical outcomes’.
• Studies that approach acceptability using only proxy terms such as feasibility, e.g., excluded if only mention being a ‘feasibility’ study.
• Studies that do not evaluate at least one surgical intervention.
• Animal studies.
• Published before the year 2000.

Systematic reviews and meta-analyses were excluded to avoid duplication of reporting; however, reference lists were checked for additional studies. Articles were limited to those written in English, or those that could be translated into English by Google translate (https://translate.google.com/).

### 2.3 Searches

A comprehensive search strategy was developed by an Information Specialist (HF). Text word searches for terms appearing in the titles and abstracts of database records were included in the strategy alongside searches of relevant subject headings. The final Ovid MEDLINE strategy was adapted with relevant subject headings (controlled vocabularies) and search syntax, appropriate to each resource (S1 Fig). No language limits were applied. A date limit of 2000 was applied.

### 2.4 Information sources

The following databases were searched on 09 November 2023: Ovid MEDLINE® ALL; Embase (via Ovid); APA PsycInfo (via Ovid); EB Health - KSR Evidence (via Ovid); Cochrane Central Register of Controlled Trials (via Wiley); International HTA database (via https://database.inahta.org/); ClinicalTrials.gov (via https://clinicaltrials.gov/); and WHO International Clinical Trials Registry Platform (via https://trialsearch.who.int/).) Results were imported into EndNote 21 (Clarivate Analytics, USA) for de-duplication.

### 2.5 Selection of sources of evidence

Results of all searches were uploaded to Covidence (http://www.covidence.org) and screened independently by two reviewers (SJ and JL, AM or AY). Full texts of potentially relevant articles were reviewed against inclusion criteria by two reviewers independently (SJ and JL, AM or AY). Disagreements were resolved through discussion.

### 2.6 Data charting process

Data extraction was performed independently by two reviewers (SJ and AM or AY) in Covidence (http://www.covidence.org). Disagreements were discussed and a final version agreed by consensus.

### 2.7 Data items

Data extracted included year and country published, study aim, methodology, disease area, description of the surgical intervention and any comparator, stage of development of the surgical intervention according to the IDEAL Framework [14], population characteristics of those reporting acceptability, definition of acceptability provided, and how and when acceptability was measured (S2 Table).

### 2.8 Critical appraisal of individual sources of evidence

The quality of studies was not assessed, which is consistent with guidance for scoping review conduct [19].

### 2.9 Analysis and presentation of the results

To address the research questions, the data extracted was mapped and tabulated by study characteristics (year published, country, methodology, surgery type, stage of development, who reported acceptability, how was acceptability measured and when was acceptability measured) and presented descriptively. The data extracted for definitions of acceptability and the content of the measurement methods provided were tabulated and narratively synthesised.

## 3. Results

### 3.1 Overall description of the included studies

**Fig 1:**
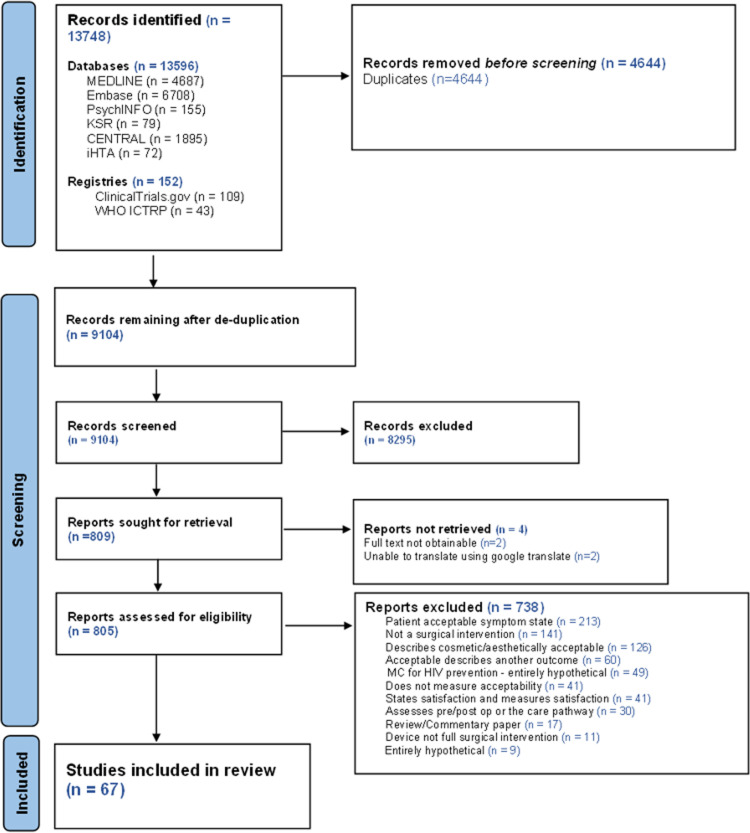
PRISMA 2020 flow diagram for reviews which included searches of databases and registers.

The searches yielded 9,104 records after de-duplication (Fig 1). Researchers screened 805 full-text articles and 67 studies were included. Fifty-three of the included studies were full publications of completed studies, six were conference abstracts of completed studies, and eight were protocols.

Of the 738 full-text articles that were excluded the main reason was that they did not assess the acceptability of the surgical intervention: 29% (n = 213) used a ‘patient acceptable symptomatic state’ which measured acceptability of symptoms, 8% (n = 60) described acceptability of another outcome measure, 7% (n = 49) reported the acceptability of male circumcision for HIV prevention and 1% (n = 8) were feasibility randomised controlled trials (RCT) assessing the acceptability of trial processes (see Fig 1. for further reasons for exclusion). Two studies were not published in English and it was not possible to translate these sufficiently using Google translate (https://translate.google.com/) and were therefore excluded.

The included studies were published between 2001 and 2023, with the highest number published in 2021 (n = 8; 12%). The included studies and were undertaken in 25 countries, with most studies taking place in the UK (n = 20; 30%) followed by India (n = 7; 10%) and the USA (n = 6; 9%). There were six studies conducted in more than one country (Table 2).

**Table 2:** Summary of key characteristics of included studies.

Study Author and year	Country	Study methodology	Clinical speciality	Elective or Trauma	Stage of devel-opment+	Population description (who reported acceptability?)	How was acceptability measured?	When was acceptability measured?^*^
***Andley et al., (2018)*** [22]	India	RCT^(CA)^	Colorectal	Elective	4	Patients receiving the surgical intervention	Questionnaire	Post-intervention
***Awoyinka et al., (2006)*** [23]	Nigeria	Cross-sectional study	Obstetrics	Elective	4	Patients receiving the surgical intervention	Questionnaire	Pre-intervention
***Banieghbal et al., (2007)*** [24]	South Africa	Retrospective case study	Gastroenterology	Elective	4	Patients receiving the surgical intervention and caregivers	Not clear	Post-intervention
***Baste et al., (2022)*** [25]	India	Cross-sectional study	Oncology	Elective	4	Patients receiving the surgical intervention	Questionnaire	Pre-intervention
***Batten et al., (2016)*** [26]	UK	RCT; Cohort study; Retrospective analysis	Orthopaedics/ Trauma	Trauma	4	Patients receiving the surgical intervention	Questionnaire	Post-intervention
***Bell et al., (2017*** [27]	UK	RCT	Rheumatology/Autoimmune diseases	Elective	1	Patients receiving the surgical intervention	Questionnaire	Post-intervention
***Bhattacharya (2014)*** [28]	UK	RCT^(P)^	Gynaecology/ Reproductive Health	Elective	4	Patients receiving the surgical intervention	Questionnaire	Post-intervention
***Bowling et al., (2008)*** [29]	UK	Cross-sectional study	Cardiology	Elective	4	Patients who may potentially receive the intervention	Questionnaire	Pre-intervention (hypothetical)
***Bunce et al., (2007)*** [30]	Lead author USA; study conducted in Tanzania	Qualitative study	Urology	Elective	4	Patients receiving the surgical intervention and patients who may potentially receive the intervention	Interviews; Focus groups	Pre- and post-intervention (both hypothetical surgery and post-surgery participants)
***Chen et al.,(2018)*** [31]	China	Non-randomised trial	Orthopaedics/ Trauma	Trauma	4	Patients receiving the surgical intervention	Questionnaire	Not clear
***Cook et al., (2018)*** [32]	UK	Systematic review, surveys, focus groups and interviews, a Delphi study and consensus meeting. ^(P)^	Orthopaedics/ Trauma	Elective	4	Patients receiving the surgical intervention, healthcare professionals (*including Surgeons*), representatives from industry, the NHS and regulatory bodies	Focus groups; survey	Post-intervention
***Cook et al.,(2020)*** [33]	UK	Qualitative study within feasibility RCT ^(P)^	Orthopaedics/ Trauma	Trauma	4	Patients who accept/ decline participation, healthcare professionals (*including Surgeons*) & trial recruiters	Interviews	Pre- and post-intervention
***Crombag et al., (2021)*** [34]	Lead author Belgium and UK affiliations	Qualitative study	Neonatal	Elective	4	Parents/patients who may potentially receive the intervention	Interviews	Pre- and post- intervention
***Deshmane et al., (2021)*** [35]	India	Cross-sectional study ^(CA)^	Oncology	Elective	4	Patients receiving the surgical intervention	Questionnaire	Post-intervention
***Dunmoye et al., (2001)*** [36]	South Africa	Observational study; retrospective survey	Urology	Elective	4	Patients receiving the surgical intervention	Interviews	Post-intervention
***Eisinger et al., (2001)*** [37]	France	Cross-sectional study	Oncology	Elective	4	Healthcare professionals (*including Surgeons*), patients who may potentially receive the intervention	Questionnaire	Pre-intervention (hypothetical)
***Ferreres et al., (2010)*** [38]	Argentina	RCT^(P) (CA)^	Gastroenterology	Elective	4	Patients receiving the surgical intervention	Questionnaire	Post-intervention
***Ficty and Teiseseyre, (2021)*** [39]	France	Cohort study	Oncology	Elective	4	Lay people and healthcare professionals (*including Surgeons*)	Interviews; Questionnaire	Pre- and post-intervention (hypothetical for laypeople; NA healthcare professionals)
***Gaba et al., (2021)*** [40]	UK	Cohort study	Oncology	Elective	4	Patients receiving the surgical intervention and patients who may potentially receive the intervention	Questionnaire	Pre- and post-intervention (depends on if patient has undergone the surgery)
***Giampaolino et al., (2016)*** [41]	Italy	RCT	Obstetrics	Elective	4	Patients receiving the surgical intervention	Questionnaire	Post-intervention
***Giannoudis et al., (2023)*** [42]	UK	Cross-sectional study	Orthopaedics/ Trauma	Trauma	3	Patients receiving the surgical intervention	Questionnaire (Survey)	Pre-intervention
***Gordon-Maclean et al., (2014)*** [43]	Lead Author UK, Study in Uganda	Cohort study	Obstetrics	Elective	4	Patients receiving the surgical intervention	Questionnaire (Survey)	Post-intervention
***Goto et al., (2016)*** [44]	Japan	Cross-sectional study ^(CA)^	Gastroenterology	Elective	4	Healthcare professionals	Questionnaire (Survey)	NA (healthcare professionals)
***Hajong and Khariong, (2016)*** [45]	India	Comparative study	Gastroenterology	Elective	4	Patients receiving the surgical intervention	Questionnaire	Post-intervention
***Handaya et al., (2020)*** [46]	Indonesia	Case study	Gynaecology/ Reproductive Health	Elective	3	Patients receiving the surgical intervention	Interviews	Post-intervention
***Harrison et al.,(2017)*** [47]	UK	Qualitative study within a feasibility RCT^(P)^	Orthopaedics/ Trauma	Elective	4	Patients receiving the surgical intervention	Interviews	Post-intervention
***Herman-Roloff et al., (2011)*** [48]	Lead author USA; study in Kenya	Qualitative study	HIV Prevention	Elective	4	Patients who may potentially receive the intervention	Focus groups	Pre-intervention (hypothetical)
***Hii et al., (2021)*** [49]	Australia	Case study; Prospectively collected data	Gastroenterology	Elective	4	Patients receiving the surgical intervention and patients who may potentially receive the intervention	Questionnaire	Post-intervention
***Holman et al., (2013)*** [50]	USA	Cross-sectional study	Oncology	Elective	4	Patients who may potentially receive the intervention	Questionnaire (Survey)	Pre-intervention (hypothetical)
***Huang et al., (2007)*** [51]	Taiwan	RCT	Colorectal	Elective	4	Patients receiving the surgical intervention	Questionnaire	Post-intervention
***Kawamura et al., (2009)*** [52]	Japan	Cohort study	Oncology	Elective	4	Patients receiving the surgical intervention	Questionnaire	Post-intervention
***Kim et al., (2023)*** [53]	Canada	Qualitative study	Obesity	Elective	4	Patients who may potentially receive the intervention	Interviews	Pre-intervention (hypothetical)
***Langford et al., (2008)*** [54]	USA	Cross-sectional study	Urology	Elective	4	Patients who may potentially receive the intervention	Questionnaire	Pre-intervention (hypothetical)
***Littlewood et al., (2021)*** [55]	UK	Qualitative study within a feasibility RCT^(P)^	Orthopaedics/ Trauma	Trauma	4	Patients receiving the surgical intervention	Interviews	Post-intervention
***Mir et al., (2014)*** [56]	Canada	Qualitative study	Oncology	Elective	4	Patients who may potentially receive the intervention (had previously had a type of surgery- asking about a further type of surgery)	Interviews	Pre-intervention (hypothetical)
***Nair et al., (2021)*** [57]	India	Cross-sectional study	Oncology	Elective	4	Patients who may potentially receive the intervention (had previously had a type of surgery- asking about a further type)	Questionnaire	Pre-intervention (hypothetical)
***Nebgen et al., (2018)*** [58]	USA	Non-randomised trial; pilot study	Oncology	Elective	4	Patients who may potentially receive the intervention	Questionnaire	Pre-intervention
***Nuccio et al., (2017)*** [59]	Ethiopia	Cohort study	Gynaecology/ Reproductive Health	Elective	4	Patients receiving the surgical intervention	Questionnaire	Post-intervention
***O’ Brien et al., (2016)*** [60]	Australia	Cohort study; prospective cohort study with comparative control data	Obesity/ Diabetes	Elective	4	Patients receiving the surgical intervention	Questionnaire	Post-intervention (recruitment and retention)
***Olakkengil et al., (2010)*** [61]	Australia	Cross-sectional study	Nephrology/ Transplantology	Elective	4	Patients receiving the surgical intervention	Questionnaire	Post-intervention (hypothesised about using a different technique for same procedure)
***Parkar et al., (2003)*** [62]	Kenya	Observational study	Obstetrics	Elective	4	Patients receiving the surgical intervention and Healthcare professionals (*including Surgeons*)	Not clear	Not clear
***Paynter et al., (2023)*** [15]	Australia	Qualitative study	Orthopaedics/ Trauma	Elective	4	Patients receiving the surgical intervention	Interviews	Post-intervention
***Penketh et al., (2006)*** [63]	UK	Observational study	Obstetrics	Elective	4	Patients receiving the surgical intervention	Not clear	Post-intervention
***Penna et al., (2014)*** [64]	UK	Cross-sectional study	Obesity	Elective	4	Healthcare professionals (*including Surgeons*)	Questionnaire (Survey)	NA (healthcare professionals)
***Pulman et al., (2015)*** [65]	Canada	Cross-sectional study	Oncology	Elective	4	Healthcare professionals (*including Surgeons*)	Questionnaire	NA (healthcare professionals)
***Qiu et al., (2011)*** [66]	China	RCT; Comparative study	Gynaecology/ Reproductive Health	Elective	4	Patients receiving the surgical intervention	Questionnaire	Post-intervention
***Retrouvey et al., (2019)*** [67]	Canada	Qualitative study	Oncology	Elective	4	Patients receiving the surgical intervention and patients who may potentially receive the intervention	Interviews	Pre-intervention (hypothetical) Post-intervention
***Rosnov (2009)*** [68]	USA	Cross-sectional study	Obesity	Elective	4	Patients who may potentially receive the intervention and their caregivers	Questionnaire	Pre-intervention (hypothetical)
***Sacco et al., (2020)*** [69]	UK and Ireland	Cross-sectional study	Foetal surgery/ Neurosurgery	Elective	4	Healthcare professionals (*including Surgeons*)	Questionnaire	NA (healthcare professionals)
***Sarwer et al., (2013)*** [70]	USA	Cross-sectional study	Obesity/ Diabetes	Elective	4	Patients receiving the surgical intervention and patients who may potentially receive the intervention	Questionnaire	Pre-intervention (hypothetical)
***Smith et al., (2022)*** [71]	UK	Cross-sectional study ^(P)^	Gastroenterology	Elective	4	Patients receiving the surgical intervention and patients who may potentially receive the intervention	Interviews; Questionnaire	Pre-intervention (including hypothetical) Post-intervention
***Smithling et al., (2018)*** [72]	Rwanda	Observational study ^(CA)^	Obstetrics	Elective	4	Patients receiving the surgical intervention and patients who may potentially receive the intervention	Interviews; Questionnaire	Pre-intervention (hypothetical)
***Sokal et al., (2014)*** [73]	USA, Zambia and Kenya	RCT	HIV prevention	Elective	4	Patients receiving the surgical intervention	Interviews; Questionnaire	Post-intervention
***Solanki et al., (2015)*** [74]	India	Observational study	Paediatrics	Elective	4	Patients receiving the surgical intervention and patients family	Not clear	Pre-intervention Post-intervention
***Soomro et al., (2022)*** [75]	UK	Qualitative study within an RCT	Urology	Elective	3	Patients receiving the surgical intervention	Interviews; Questionnaire; Other: Screening logs	Post-intervention
***Srikesavan et al., (2021)*** [76]	UK	Qualitative study within an RCT	Orthopaedics/ Trauma	Elective	4	Patients receiving the surgical intervention (trial participants) and healthcare professionals (*including Surgeons*)	Interviews	Post-intervention
***Stanford et al., (2015)*** [77]	USA	Cross-sectional study	Obesity	Elective	4	Screened general population, further questions for patients who may potentially receive the intervention	Questionnaire (Survey)	Pre-intervention (hypothetical).
***Straiton et al., (2022)*** [78]	Australia	Qualitative study ^(CA)^	Cardiology	Elective	4	Patients receiving the surgical intervention and their caregivers	Interviews	Post-intervention
***Summers et al., (2014)*** [79]	UK	Qualitative study	Obesity/ Diabetes	Elective	4	Patients receiving the surgical intervention	Interviews	Pre-intervention (hypothetical)
***Thaha et al., (2009)*** [80]	UK	RCT	Colorectal	Elective	4	Patients receiving the surgical intervention	Questionnaire	Post-intervention
***Turner et al., (2015)*** [81]	UK	Qualitative study; interviews within a UK cohort study	Obesity/ Diabetes	Elective	4	Patients receiving the surgical intervention	Interviews	Pre-intervention (hypothetical)
***Tyagi et al., (2012)*** [82]	UK	Cross-sectional study ^(CA)^	Gynaecology/ Reproductive Health	Elective	4	Healthcare professionals	Questionnaire	NA (healthcare professionals)
***van Geelen et al., (2013)*** [83]	Netherlands	Qualitative study	Obesity	Elective	4	Healthcare professionals, parents and patients who may potentially receive the intervention	Interviews	Pre-intervention (hypothetical)
***Vartanian et al., (2009)*** [84]	Brazil	Case study	Oncology	Elective	4	Patients receiving the surgical intervention	Interviews; Questionnaire	Post-intervention
***Wagg et al., (2017)*** [85]	Finland	Cross-sectional study ^(CA)^	Gynaecology/ Reproductive Health	Elective	4	Patients who may potentially receive the intervention	Questionnaire	Pre-intervention (hypothetical)
***Wong et al., (2023)*** [86]	UK	Qualitative study within a feasibility RCT^(P)^	Urology	Elective	4	Patients receiving the surgical intervention	Interviews	Pre-intervention Post-intervention
***Yasir et al., (2009)*** [87]	India	Cohort study; Prospective study	Colorectal	Elective	4	Patients receiving the surgical intervention	Not clear	Not clear

* Timing definitions: Pre-intervention delivery (i.e., prior to any exposure to the intervention (prospective/ forward-looking). Post-intervention delivery (i.e., following completion of the intervention or at the end of the intervention delivery period when no further exposure is planned).

+ Stage of development according to the IDEAL recommendations(14) was assigned by two independent reviewers based on the following definitions; Stage 0 – The Preclinical Stage, Stage 1 - Idea Deals with proof of concept, involving first use in humans. Stage 2a - Development The technical details are refined and stabilised through experience in a small case series, Stage 2b - Exploration A common understanding of the procedure is reached among operators in a multi-centre study, and the obstacles to a definitive comparative trial are addressed. Stage 3 - Assessment Typically a randomized controlled trial (RCT). Stage 4 - Long-term study Surveillance to identify rare and late outcomes as well as a possible broadening of ‘accepted’ indications.

(P) = Protocol ^(CA)^ = Conference abstract

**Fig 2:**
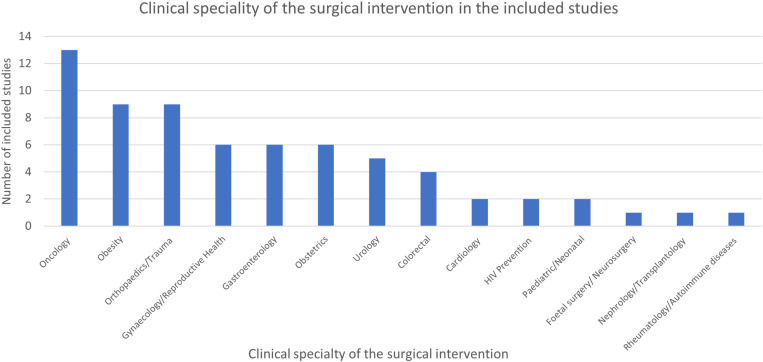
Clinical speciality of the surgical intervention in the included studies.

The most common clinical speciality for the surgical intervention was oncology (n = 13) followed by obesity (n = 9) and orthopaedics/trauma (n = 9) (Fig 2). The majority (92.5%) of studies investigated the acceptability of an elective surgical intervention (as opposed to a trauma surgical intervention).

### 3.2 Defining acceptability

Twenty-four (36%) of the 67 included studies used the term acceptability in their title and 36 (54%) as part of their main study aim, with 17 (25%) using the term in both the title and within the aim.

Seven (10%) of the included studies provided an explicit conceptual definition of acceptability, with six of these (85%) utilising or referencing the definition provided in the TFA [6] (Table 3).

**Table 3:** Conceptual definitions used by the studies who provided this information.

Study author	Definition provided
***Crombag et al.,*** ***(2021)*** [34]	*‘Acceptability can be defined as a construct that reflects the extent to which people receiving a healthcare intervention consider it appropriate, based on anticipated or experienced responses to the intervention.’ (TFA)*
***Paynter et al., (2023)*** [15]	*‘Acceptability is a multi-faceted construct that reflects the extent to which people delivering or receiving a healthcare intervention consider it to be appropriate, based on anticipated or experienced cognitive and emotional responses to the intervention.’ (TFA)*
***Rosnov*** ***(2009*)** [68]	*‘Define treatment acceptability as judgments by laypersons, clients, and others of whether treatment procedures are appropriate, fair, and reasonable for the problem or client.’ (Kazdin 1981)*
***Sacco et al., (2020)*** [69]	*‘Acceptability is a key consideration in the implementation of new healthcare interventions such as foetal surgery, for both providers and receivers. Buy in from healthcare professionals referring, treating or caring for patients is necessary to ensure that the intervention is offered and delivered as intended.’ (TFA)*
***Soomro et al., (2022)*** [75]	*‘….the acceptability of healthcare interventions based on work by Sekhon et al., (2022)’ (TFA)*
***Straiton et al., (2022)*** [78]	*‘Theoretical Framework of Acceptability.’*
***Wong et al., (2023)*** [86]	*‘Theoretical Framework of Acceptability.’*

### 3.3 Measuring acceptability

#### 3.3.1 Study design.

The included studies utilised different methods and procedures to collect and analyse the data on acceptability. The most common study design used was a cross sectional design (using a questionnaire) (n = 20; 30%), qualitative methods (n = 10; 15%) and RCTs in which acceptability of surgery was measured as one of the study outcomes (n = 9; 13%). Six (9%) were embedded qualitative studies to assess acceptability of a surgical intervention within an RCT or feasibility RCT (Table 2).

Over half of the included studies reported the acceptability of a single surgical intervention (n = 38; 57%), 19 (28%) reported the acceptability in a study comparing two or more surgical interventions and eight (12%) considered surgical vs non-surgical interventions.

#### 3.3.2 Who reported acceptability?

Thirty-three studies (49%) asked patients who had received the surgical intervention to report acceptability, whilst 9 studies (13%) asked patients to report acceptability of a possible future surgical intervention. Seven studies (10%) asked both patients who had received and those who may receive the surgical intervention to report acceptability. Five studies (7%) reported acceptability of the intervention to both patients and surgeons, and a further three (4%) reported acceptability to surgeons (further detail of who reported acceptability in all included studies is provided in Table 2).

Of the 59 studies that were complete, 49 (83%) provided the age of the participants in the study (the age of the participants ranged from 20 to 84 years), 46 (79%) provided information on the gender of the participants (across the included studies, 24% of the participants were male and 76% of the participants were female) and 12 (20%) of the studies reported ethnicity of the participants who had reported acceptability (where this was reported it was most predominantly described as ‘British’/ 'White British’/ 'White’/ 'Caucasian’).

#### 3.3.3 When was acceptability measured?

Thirty studies (45%) measured acceptability after the surgical intervention had taken place, 20 studies (30%) collected data on acceptability before the surgical intervention had taken place, with 16 of these 20 studies (80%) asking patients to report acceptability of a possible future surgical intervention. Nine studies (13%) measured acceptability both before the surgical intervention and after the surgical intervention had taken place and for three studies it was unclear when the measurement had taken place in relation to the surgical intervention (Table 2).

To explore at what stage in the surgical interventions development the acceptability was measured, all studies were classified according to the IDEAL Framework [14]. Sixty-three studies were Stage 4 (which is described as surveillance to identify rare and late outcomes as well as a possible broadening of ‘accepted’ indications), three studies were Stage 3, (described as evaluation of the intervention against current practice) and one study was Stage 1 (described as proof of concept, involving first use in humans).

#### 3.3.4 How was data on acceptability collected?

**Fig 3:**
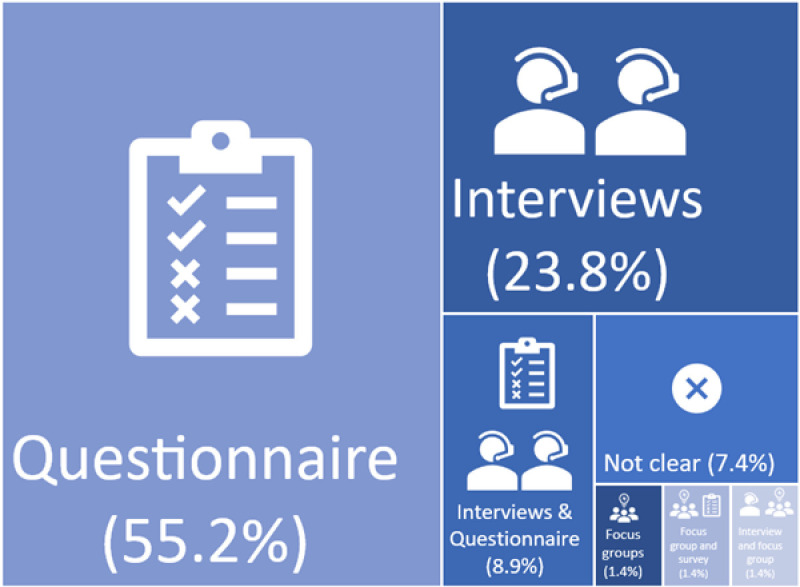
Data collection methods used to obtain information about acceptability of the surgical intervention in the included studies.

Most studies (n = 62; 92%) provided a description of the data collection methods, with the most common being a questionnaire used to collect data regarding acceptability of the surgical intervention (n = 37; 55.2%) followed by qualitative interviews (n = 16; 23.8%) (Fig 3).

Of the 43 studies utilising questionnaires, (comprising of n = 37 studies that used a questionnaire only to collect data on acceptability and n = 6 studies that used a combination of both a questionnaire and qualitative interviews), 40 (93%) provided details of the questionnaire or questions used and it was unclear on the remaining three (S3 Table). Of the 40 studies that provided some detail, 30 (73%) reported using bespoke questions or a combination of scales to measure acceptability; five (17%) of these reported at least some information about the validity and reliability of the measure. Eleven studies (27%) that used a questionnaire reported using or adapting an existing questionnaire or using a combination of existing questionnaires, and six (55%) of these 11 provided information about the validity and reliability of the acceptability measure that was used.

Of the 25 studies that undertook qualitative interviews or focus groups (comprising of n = 16 studies that conducted qualitative interviews alone, n = 2 studies conducting focus groups, n = 6 studies conducting both qualitative interviews and using a questionnaire and n = 1 study conducted both interviews and focus groups), 20 studies (80%) gave further details of the methods of exploring acceptability, with six studies (29%) of these 20 specifically mentioning a topic guide (S4 Table).

Within the measurement of acceptability, eight studies (12%) used (or included as a component of the measure) the concept of satisfaction. In addition, seven studies (10%) included pain scores, seven (10%) cosmesis/cosmetic appearance assessment and six (9%) used ‘would you recommend the intervention to family or friends’.

## 4. Discussion

### 4.1 Summary of evidence

This scoping review sought to map the existing evidence on how studies evaluating the acceptability of a surgical intervention define, measure and report acceptability. The findings highlighted a lack of clarity and consistency in the definition and measurement of acceptability. The vast majority (n = 60; 90%) of the included studies did not provide a definition of acceptability, despite 25% (n = 17) of them using the term in both their title and aim. This is an increase compared to a previous study which identified systematic reviews that claim to define, theorise or measure acceptability of healthcare interventions and reported from 43 reviews included, none explicitly theorised or defined acceptability [6].

Where a conceptual definition of acceptability was provided by a study, it was most frequently the Theoretical Framework of Acceptability definition [6]; although this conceptual definition was only used in a very small number of studies overall.

Studies assessed acceptability using a variety of qualitative and quantitative methods. This variation reflects the current lack of standardisation in measurement of acceptability in evaluations of surgical interventions. A standardised conceptualisation and measurement instrument for acceptability of a surgery is a necessary step to being able to report and improve acceptability, enhancing clinical decision-making, and enriching patient experiences. Development of a new measure should involve evaluation of the strengths and limitations of the various methods used to date. Providing patients with clearer information on acceptability will help in making informed decisions about surgical treatments, promoting patient-centered care and better health outcomes.

Terms and concepts associated with acceptability were also used by some of the included studies. For example, eight studies reported measuring acceptability, but used satisfaction measures either solely or as part of the assessment. This finding is similar to an overview of reviews identifying studies that claimed to define, theorise or measure the acceptability of healthcare interventions. In this overview 50% of the identified reviews using self-report assessments of acceptability included a measure of satisfaction [6]. This suggests a knowledge gap as to whether concepts used in the assessment of acceptability, such as satisfaction, are separate to acceptability, or whether they form components of acceptability.

Over 90% of the included studies assessed the acceptability of an elective surgical intervention, as opposed to a trauma or emergency surgical intervention. It is possible that the definition of acceptability differs depending on if a surgery is elective or related to trauma or emergency and on the clinical speciality, which should also be considered in future research.

Most studies (73%) asked patients who had undergone, or may potentially undergo the surgical intervention to report acceptability, reflecting the known importance of patient reported outcome measures in understanding patient perspectives [88], engaging patients in co-evaluation of their surgery [89] and capturing what matters to them [90]. However, overall the population characteristics of those reporting acceptability were not well reported. It is suggested that age, gender, ethnicity, cultural and other socio-economic factors may influence the acceptability of a surgical intervention [67], therefore, it would be beneficial to examine how these characteristics and any other systemic differences, affect how acceptability is reported*.* It would also be valuable to explore if surgeon and patient perspectives should be considered separately, or if it is feasible to combine different stakeholder responses in a single outcome measure to gather comprehensive information about acceptability of surgical interventions [91].

The majority (98.5%) of included studies were investigating an already established surgical intervention (described in this scoping review using the IDEAL framework [14]), yet it is suggested that the development stage of a surgical intervention can affect its acceptability [56]. The guidance and workstreams specifically for safely developing, refining and testing novel surgical procedures during their early-stages of development are in their infancy [92,93] which may account for why only one study reported acceptability at this very early-stage of the surgical interventions development.

There was further variability demonstrated in the timing of when acceptability was assessed. There were 30 studies (45%) that measured acceptability after the surgical intervention and 20 studies (30%) that measured acceptability before the surgical intervention. It has been suggested that different factors influence prospective (i.e., anticipated) and retrospective (i.e., experienced) acceptability and that acceptability should be assessed prior to the patient experiencing an intervention wherever possible [6,8,29]. Additionally, measuring acceptability early in the surgical intervention’s development [14], would allow improvements in acceptability to be made, and therefore improving patient care, and health outcomes. Future research should consider when the optimal time is to measure acceptability of a surgical intervention.

### 4.2 Strengths and Limitations

This scoping review included rigorous database searches, and mapped the evidence on how the acceptability of surgical interventions is currently defined and measured in evaluation research [94]. The protocol was written and made publicly available prior to the searches being undertaken. Study selection and data extraction were undertaken independently by two reviewers. The included studies were undertaken across 25 countries, highlighting that acceptability is an internationally utilised concept, and has wide relevance. The concept of acceptability may be considered differently in different languages or countries, which may in turn affect the definition and measurement of it [6].

While the searches were designed to be comprehensive, relevant literature assessing acceptability using alternative terminology or varied interpretations of the word acceptability could have been missed. It was beyond the scope of this project to include search terms for patient or surgeon preferences, perspectives, satisfaction, or for proxy questions such as ‘would you recommend this to your friends and family’ due to the number of results that this would generate. We focused on studies that reported acceptability in the title or abstract (specifically, only records with ‘acceptab*’ in the title or abstract were searched for) on the assumption that these studies would provide the richest data source for how surgical intervention acceptability is being defined and measured within the field.

## 5. Conclusion

Despite searching across all types of surgery over a twenty-three year period, we identified only a small pool of studies reporting measuring acceptability of a surgical intervention in the title or abstract. Studies often did not provide a definition of acceptability and there was a lack of clarity in the use of the concept. There was also substantial variability in how and when acceptability was measured and from which perspective.

Further research is required to explore the most appropriate approaches to promote a more consistent conceptualisation and accurate measurement by addressing the variability in whether, how and when acceptability is considered in the evaluation of surgical interventions. This should include development of consensus on core components of acceptability for a surgical intervention, with consideration of the stage of surgical innovation, measurement tools and implementation of good practice.

## Supporting information

S1 Table PRISMA-ScR Checklist. (DOCX)

S2 Table Copy of Covidence Data extraction form. (DOCX)

S3 Table Details provided on the questionnaires of questions used to collect data on acceptability. (DOCX)

S4 Table Details provided by the studies utilising interviews/ focus groups on the questions used to collect data on acceptability. (DOCX)

S1 Fig Search strategies. (PDF)
