# Supplementary material for: Defining and measuring acceptability of surgical interventions: A scoping review
Source: PLoS One. 2025 Jun 3;20(6):e0323738. doi: 10.1371/journal.pone.0323738 (PMC12132998; doi:10.1371/journal.pone.0323738)
Supplement: S2 Table — (DOCX) [file pone.0323738.s002.docx]

**S2 Table: Copy of COVIDENCE DATA EXTRACTION FORM**

| Title of paper / abstract / report that data are extracted from |
| --- |
| Lead author name |
| Year published |
| Country in which the study was conducted |
| Characteristics of included studies |
| Methods |
| Aim of study |
| Type of study |
| Disease area |
| Surgical Intervention description |
| Details of the comparator and details of these (e.g. surgery vs surgery, surgery vs placebo, surgery vs non-surgical intervention) |
| Trauma or elective surgical intervention |
| What stage of development is the surgical intervention at?(using the IDEAL stages)  Stage 0 – The Preclinical Stage  Stage 1 - Idea Deals with proof of concept, involving first use in humans.  Stage 2a - Development The technical details are refined and stabilised through experience in a small case series.  Stage 2b - Exploration A common understanding of the procedure is reached among operators in a multi-centre study, and the obstacles to a definitive comparative trial are addressed.  Stage 3 - Assessment Typically a randomized controlled trial (RCT).  Stage 4 - Long-term study Surveillance to identify rare and late outcomes as well as a possible broadening of ‘accepted’ indications. |
| Participants |
| Population description (who is reporting Acceptability?) |
| Age of participants reporting acceptability of the surgical intervention |
| Gender of participants reporting acceptability |
| Ethnicity of participants reporting acceptability |
| Total number of participants who reported acceptability |
| Was a definition of acceptability provided? (or able to determine a definition that was used) Yes/No |
| If yes, what was the definition |
| How was acceptability measured |
| If a Questionnaire was used to measure acceptability what was it called? (N/A if no questionnaire used) |
| If an interview/focus groups, what specific questions were asked about acceptability? (N/A if no interview/focus groups) |
| When was acceptability measured?  Pre-intervention delivery (i.e. prior to any exposure to the intervention (prospective/ forward-looking)  During intervention delivery (i.e. concurrent assessment of acceptability; when there has been some degree of exposure to the intervention  Post-intervention delivery (i.e. following completion of the intervention or at the end of the intervention delivery period when no further exposure is planned). |
| Does the study provide any information about how the acceptability measure performs e.g. reliability |
| Does the study provide any other relevant information about acceptability |
| Study funding sources |
