# Supplementary material for: Defining and measuring acceptability of surgical interventions: A scoping review
Source: PLoS One. 2025 Jun 3;20(6):e0323738. doi: 10.1371/journal.pone.0323738 (PMC12132998; doi:10.1371/journal.pone.0323738)
Supplement: S4 Table — (DOCX) [file pone.0323738.s004.docx]

**S4 Table: Details provided by the studies utilising interviews/ focus groups on the questions used to collect data on acceptability**

| Study author | Details provided on the interview/focus group questions |
| --- | --- |
| *Bunce et al., (2007)*[30] | Semi-structured, open-ended data collection instruments used to ensure consistency of questions. Focus group and interview guides were made up of three main question domains - participant characteristics, contraceptive decision making and perceptions of reproductive health programs. Respondents were asked the same questions in the same sequence; inductive probing on key responses was conducted. |
| *Cook et al., (2018)*[32]*^#^* | Using focus groups to access a broad range of stakeholder views and opinions on the acceptability of the use of patches in the augmentation of rotator cuff repair and the trial design options that may be used to test them. Themes and issues identified from the surgeon survey will help to form topics for discussion if findings are available. During the focus group, the project will be introduced and participants asked to consider a number of relevant issues, scenarios or vignettes. Key items of information about the possible trial design options, the choice of study arms, most appropriate outcome measures and methods of data collection (e.g. biopsy, patient questionnaires, site visits), will be raised. |
| *Cook et al.,(2020)*[33] | A qualitative study will explore trial participants and surgeons’ views and experiences of the intervention and trial processes. Flexible interview schedule will be developed following discussions with the research team, PPI team and surgeons with expertise. Will discuss the impact and acceptability of the intervention and clinical follow-up within the context of the daily lives of patients and their families to assess the interplay between the clinical intervention and their individual circumstances, for example, employment, housing and family composition. Surgeons will be asked about the feasibility/acceptability of providing either intervention on a regular basis, willingness to randomise, workload/staffing implications, training requirements, and readiness to employ the findings of a definitive trial into their normal practices |
| *Crombag et al.,(2021)*[34] | All interviews started with an open question inviting participants to share their thoughts, views, feelings and experiences whether to participate or not in MFS. The seven framework components (of TFA) were used as prompts to further explore the different levels of the decision (Interview guide available). |
| *Dunmoye et al.,(2001)*[36] | Awareness of availability of vasectomy procedure through health personnel either in or outside of a family planning clinic / friends / wives / media. Regrets of having the procedure. Recommend to friends / family |
| *Ficty and Teiseseyre, (2021)*[39]^#^ | To explore the factors involved in the judgments, as advocated by Anderson (2001) and as done Mullet & Girard (2000) With an interviewer a set of cards were arranged randomly and in a different order for each participant. The question “how acceptable do you consider this therapeutic proposal?” Participants responded by marking a 35-cm line somewhere between the left-hand anchor of “Not at all acceptable” and the right-hand anchor of “completely acceptable”. |
| *Handaya et al.,(2020)*[46] | No detail of exact questions asked re acceptability - Patients underwent personal interviews to assess their current conditions assess the postoperative results, social acceptance of the procedure, and sexual satisfaction using the Female Sexual Function Index. |
| *Harrison et al.,(2017)*[47] | The guide will focus on their experiences of living with Dupuytren’s contracture pre and post intervention, previous experiences of treatment, recovery post intervention, views on the treatment received, the suitability and ease of understanding and completing the hand function outcome measures, and their reflections on participating in the trial. |
| *Herman-Roloff et al., (2011)*[48] | The Focus Group Discussion guide consisted of 12 open-ended questions with probes about (the surgery) uptake and acceptability |
| *Kim et al., (2023)*[53] | No detail of exact questions asked re acceptability - Participants were asked about their diagnosis, perceptions towards weight and fertility, and attitudes towards surgery as a treatment option (using the theory of reasoned action and the transtheoretical model). |
| *Littlewood et al., (2021)*[55] | The interviews will be based on semi-structured topic guides developed in relation to the pre-specified aims but also with our patient and public involvement group (approximately 30-min telephone interview). It is expected that approximately 20 patients will be sufficient to attain rich data. |
| *Mir et al., (2014)*[56] | Patients were presented with hypothetical scenarios including a scenario in which the surgery was offered to them routinely and a scenario in which the surgery was in a clinical trial. |
| *Paynter et al.,(2023)*[15] | Interviews were structured using an interview guide based on the TFA. Questions devised to address each of the TFA constructs in turn. In the context of elective surgery, members of the research team predicted that a question regarding ethicality may not elicit much discussion. Nonetheless, a question was included to address all constructs of the TFA. The interview concluded with a general (nonspecific) question about acceptability (Can I ask you now to think about overall, when considering all the things you've spoken about, what do you think about your joint replacement experience) as recommended by Sekhon et al. (2017). During development of the interview guide, considering the risk profile associated with surgical interventions, an additional question to elicit perceptions about safety and risk of the intervention was generated and added to the interview guide. This question was asked towards the end of the interview to reduce any potential contamination of responses to the TFA- based questions. |
| *Retrouvey et al., (2019)*[67] | The research team developed an interview guide thorough a detailed review of the literature and access to care frameworks. The interviews discussed womens’ experience with cancer, their views and opinions about (the surgery) and their experience trying to access (the surgery). |
| *Smith et al., (2022)*[71]*^#^* | Semi-structured interviews - Topic guides and preprepared questions will be developed by the interviewers, with input from stakeholders, and will be used to ascertain participants views on risk-predictive models, along with acceptability of prophylactic mesh and factors that might make it more acceptable |
| *Smithling et al., (2018)*[72]*^#^* | No detail of exact questions asked re acceptability - The participants then completed a series of open- and close-ended questions. |
| *Sokal et al., (2014)*[73]*^#^* | Recommendation to friends and satisfaction with cosmetic results. |
| *Soomro et al., (2022)*[75]*^#^* | Interview topic guides will be developed, covering aspects of trial rationale, design and conduct with a specific focus on illuminating the influences on trial recruitment and processes linked to the pathway (specifically exploring barriers and facilitators within local contexts). For those participants who declined trial participation we will investigate whether there were specific aspects of trial design or conduct that led to their decision to not to be involved. Data collection and analysis will be informed by the Theoretical Domains Framework and/or the Theoretical Framework of Acceptability, which has been applied in existing studies exploring trial feasibility in other contexts (Sekhon et al, 2017). |
| *Srikesavan et al., (2021)*[76] | No detail of exact questions asked re acceptability - Acceptability reported in a section of results with the sub heading 'Acceptability: treatment satisfaction and improved outcomes' for trial participants. Surgeons and physios were asked about trial participants acceptability 'However, surgeons and physiotherapists felt that trial participants found all the treatment arms acceptable once they were in the trial.' |
| *Straiton et al., (2022)*[78] | Assessed outcomes (symptom relief), risks/ benefits, regrets. |
| *Summers et al., (2014)*[79] | Interview guide developed by the study team in consultation with two lay advisors. Open-ended questions relating to one of four topic areas: diabetes and diabetes management, weight and weight management, weight loss surgery and participating in weight loss research was discussed towards the end of the interview, and in order to ensure that participants were informed enough to comment on their willingness to consider surgery, interviewees were provided with information on the advantages and disadvantages of surgery. To capture participants pre-interview stance, interviewees were first asked about their knowledge of and willingness to consider surgery before any information was given. This was then re-evaluated once participants had been given the information. |
| *Turner et al., (2015)*[81] | A topic guide was used to ensure consistency across the interviews. It covered the following areas: participants views and experiences of Type 2 diabetes; how they managed their diabetes; what factors affected treatment concordance; what they saw as the advantages and disadvantages of different treatment options (i.e. lifestyle modification, oral medication, injections, weight loss surgery); and what treatment characteristics and outcomes they valued and would like in any future treatments. |
| *van Geelen et al., (2013)*[83] | Researchers collected detailed information on the participants views and experiences through semi-structured interviews, with a list of open questions; what do you think of bariatric surgery, especially gastric banding, as a treatment for morbid obesity? What kind of risks would you find acceptable? Would you want it for your own child? If yes, why? If no, why not? Would you want it for yourself? If yes, why? If no, why not? Do you think that your child would want it? If yes, why? If no, why not? Would you recommend it to a friend? If yes, why? If no, why not? |
| *Vartanian et al., (2009)*[84]*^#^* | No detail of exact questions asked re acceptability, only - the patients were interviewed by a trained nurse. |
| *Wong et al., (2023)*[86] | The research team will carry out 12 in-depth interviews. Using the Theoretical Framework of Acceptability, affective attitudes, burden, ethicality, intervention coherence, opportunity costs and perceived effectiveness will be assessed. |

# = used both questionnaire and interview/focus group
