# Supplementary material for: Defining and measuring acceptability of surgical interventions: A scoping review
Source: PLoS One. 2025 Jun 3;20(6):e0323738. doi: 10.1371/journal.pone.0323738 (PMC12132998; doi:10.1371/journal.pone.0323738)
Supplement: S1 Fig — (PDF) [file pone.0323738.s005.pdf]

Full search strategies

Ovid MEDLINE(R) ALL

via Ovid http://ovidsp.ovid.com/

Date range searched: 1946 to November 08, 2023

Date searched: 9th November 2023

Records retrieved: 4687

1 exp *Surgical Procedures, Operative/ (2291336)

2 su.fs. (2277063)

3 (surgical* or surgery or surgeries).ti. (738386)

4 (surgical* or surgery or surgeries).ab. /freq=2 (920291)

5 or/1-4 (3908283)

6 acceptab*.ti. (13710)

7 (acceptab* adj (surgical* or surgery or surgeries or operat* or procedur* or treat* or technique* or intervention* or trial* or excis* or resect* or transplant* or graft*)).ab. (3933)

8 (acceptab* adj (perisurg* or peri-surg* or perioperat* or peri-operat* or intraop* or intra-op* or midoperat* or mid-operat* or midsurg* or mid-surg*)).ab. (345)

9 (acceptab* adj (patient* or inpatient* or outpatient* or person* or people or adult* or m?n or wom?n or p?ediatric* or child* or baby or babies or infant* or juvenil* or adoles* or youth*)).ab. (484)

10 (acceptab* adj ((healthcare or medical) adj (professional* or personnel or staff* or worker*))).ab. (2)

11 (acceptab* adj (surgeon* or doctor* or primary care provider* or general practitioner* or GP* or physician* or specialist* or nurse* or therapist*)).ab. (47)

12 or/6-11 (18300)

13 (acceptab* adj4 (surgical* or surgery or surgeries or operat* or procedur* or treat* or technique* or intervention* or trial* or excis* or resect* or transplant* or graft*)).ab. (23720)

14 (acceptab* adj4 (perisurg* or peri-surg* or perioperat* or peri-operat* or intraop* or intra-op* or midoperat* or mid-operat* or midsurg* or mid-surg*)).ab. (632)

15 (acceptab* adj4 (patient* or inpatient* or outpatient* or person* or people or adult* or m?n or wom?n or p?ediatric* or child* or baby or babies or infant* or juvenil* or adoles* or youth*)).ab. (21329)

16 (acceptab* adj4 ((healthcare or medical) adj (professional* or personnel or staff* or worker*))).ab. (148)

17 (acceptab* adj4 (surgeon* or doctor* or primary care provider* or general practitioner* or GP* or physician* or specialist* or nurse* or therapist*)).ab. (1630)

18 acceptab*.ab. /freq=2 (37772)

19 or/13-17 (43716)

20 19 and 18 (12053)

21 12 or 20 (26726)

22 5 and 21 (5743)

23 exp animals/ not humans/ (5168767)

24 22 not 23 (5670)

25 limit 24 to yr="2000 -Current" (4687)

Key:

/ = indexing term (Medical Subject Heading: MeSH)

exp = exploded indexing term (MeSH)

* before an indexing term = focussed subject heading

* = truncation

? = wildcard for 0-1 letters

ti,ab = terms in either title or abstract fields

su.fs = surgery as a floating subheading

adj3 = terms within three words of each other (any order)

/freq=2 = terms occur a frequency of 2 or more times

Embase

via Ovid http://ovidsp.ovid.com/

Date range searched: 1974 to 2023 November 07

Date searched: 9th November 2023

Records retrieved: 6708

1 exp *surgery/ (2777577)

2 su.fs. (2386510)

3 (surgical* or surgery or surgeries).ti. (838290)

4 (surgical* or surgery or surgeries).ab. /freq=2 (1368641)

5 or/1-4 (4779928)

6 acceptab*.ti. (16992)

7 (acceptab* adj (surgical* or surgery or surgeries or operat* or procedur* or treat* or technique* or intervention* or trial* or excis* or resect* or transplant* or graft*)).ab. (5741)

8 (acceptab* adj (perisurg* or peri-surg* or perioperat* or peri-operat* or intraop* or intra-op* or midoperat* or mid-operat* or midsurg* or mid-surg*)).ab. (555)

9 (acceptab* adj (patient* or inpatient* or outpatient* or person* or people or adult* or m?n or wom?n or p?ediatric* or child* or baby or babies or infant* or juvenil* or adoles* or youth*)).ab. (770)

10 (acceptab* adj ((healthcare or medical) adj (professional* or personnel or staff* or worker*))).ab. (2)

11 (acceptab* adj (surgeon* or doctor* or primary care provider* or general practitioner* or GP* or physician* or specialist* or nurse* or therapist*)).ab. (52)

12 or/6-11 (23796)

13 (acceptab* adj4 (surgical* or surgery or surgeries or operat* or procedur* or treat* or technique* or intervention* or trial* or excis* or resect* or transplant* or graft*)).ab. (33301)

14 (acceptab* adj4 (perisurg* or peri-surg* or perioperat* or peri-operat* or intraop* or intra-op* or midoperat* or mid-operat* or midsurg* or mid-surg*)).ab. (1006)

15 (acceptab* adj4 (patient* or inpatient* or outpatient* or person* or people or adult* or m?n or wom?n or p?ediatric* or child* or baby or babies or infant* or juvenil* or adoles* or youth*)).ab. (31631)

16 (acceptab* adj4 ((healthcare or medical) adj (professional* or personnel or staff* or worker*))).ab. (219)

17 (acceptab* adj4 (surgeon* or doctor* or primary care provider* or general practitioner* or GP* or physician* or specialist* or nurse* or therapist*)).ab. (2210)

18 acceptab*.ab. /freq=2 (49912)

19 or/13-17 (62854)

20 19 and 18 (16498)

21 12 or 20 (35465)

22 5 and 21 (7861)

23 (animal/ or animal experiment/ or animal model/ or animal tissue/ or nonhuman/) not exp human/ (6843344)

24 22 not 23 (7792)

25 (letter or comment*).ti. (238599)

26 (comment or letter or editorial or note).pt. (3042454)

27 or/25-26 (3105276)

28 24 not 27 (7611)

29 limit 28 to yr="2000 -Current" (6708)

Key:

/ = indexing term (Emtree Subject Heading)

exp = exploded indexing term (Emtree)

* before an indexing term = focussed subject heading

* = truncation

? = wildcard for 0-1 letters

ti,ab = terms in either title or abstract fields

su.fs = surgery as a floating subheading

adj3 = terms within three words of each other (any order)

/freq=2 = terms occur a frequency of 2 or more times

APA PsycInfo

via Ovid http://ovidsp.ovid.com/

Date range searched: 1806 to October Week 5 2023

Date searched: 9th November 2023

Records retrieved: 155

1 exp Surgery/ (55995)

2 (surgical* or surgery or surgeries).ti. (11213)

3 (surgical* or surgery or surgeries).ab. /freq=2 (19426)

4 or/1-3 (64034)

5 acceptab*.ti. (4597)

6 (acceptab* adj (surgical* or surgery or surgeries or operat* or procedur* or treat* or technique* or intervention* or trial* or excis* or resect* or transplant* or graft*)).ab. (560)

7 (acceptab* adj (perisurg* or peri-surg* or perioperat* or peri-operat* or intraop* or intra-op* or midoperat* or mid-operat* or midsurg* or mid-surg*)).ab. (0)

8 (acceptab* adj (patient* or inpatient* or outpatient* or person* or people or adult* or m?n or wom?n or p?ediatric* or child* or baby or babies or infant* or juvenil* or adoles* or youth*)).ab. (116)

9 (acceptab* adj ((healthcare or medical) adj (professional* or personnel or staff* or worker*))).ab. (0)

10 (acceptab* adj (surgeon* or doctor* or primary care provider* or general practitioner* or GP* or physician* or specialist* or nurse* or therapist*)).ab. (11)

11 or/5-10 (5172)

12 (acceptab* adj4 (surgical* or surgery or surgeries or operat* or procedur* or treat* or technique* or intervention* or trial* or excis* or resect* or transplant* or graft*)).ab. (5233)

13 (acceptab* adj4 (perisurg* or peri-surg* or perioperat* or peri-operat* or intraop* or intra-op* or midoperat* or mid-operat* or midsurg* or mid-surg*)).ab. (1)

14 (acceptab* adj4 (patient* or inpatient* or outpatient* or person* or people or adult* or m?n or wom?n or p?ediatric* or child* or baby or babies or infant* or juvenil* or adoles* or youth*)).ab. (3997)

15 (acceptab* adj4 ((healthcare or medical) adj (professional* or personnel or staff* or worker*))).ab. (15)

16 (acceptab* adj4 (surgeon* or doctor* or primary care provider* or general practitioner* or GP* or physician* or specialist* or nurse* or therapist*)).ab. (372)

17 acceptab*.ab. /freq=2 (11229)

18 or/12-16 (8841)

19 18 and 17 (3878)

20 11 or 19 (7546)

21 4 and 20 (169)

22 limit 21 to yr="2000 -Current" (155)

Key:

/ = indexing term (American Psychological Association's Thesaurus of Psychological Index Terms)

exp = exploded indexing term

* = truncation

? = wildcard for 0-1 letters

ti,ab = terms in either title or abstract fields

adj3 = terms within three words of each other (any order)

/freq=2 = terms occur a frequency of 2 or more times

EB Health - KSR Evidence

via Ovid http://ovidsp.ovid.com/

Date range searched: 2015 to 2023 Week 45

Date searched: 9th November 2023

Records retrieved: 79

1 (surgical* or surgery or surgeries).ti. (13587)

2 (surgical* or surgery or surgeries).ab. /freq=2 (22412)

3 or/1-2 (25216)

4 acceptab*.ti. (396)

5 (acceptab* adj (surgical* or surgery or surgeries or operat* or procedur* or treat* or technique* or intervention* or trial* or excis* or resect* or transplant* or graft*)).ab. (134)

6 (acceptab* adj (perisurg* or peri-surg* or perioperat* or peri-operat* or intraop* or intra-op* or midoperat* or mid-operat* or midsurg* or mid-surg*)).ab. (16)

7 (acceptab* adj (patient* or inpatient* or outpatient* or person* or people or adult* or m?n or wom?n or p?ediatric* or child* or baby or babies or infant* or juvenil* or adoles* or youth*)).ab. (15)

8 (acceptab* adj ((healthcare or medical) adj (professional* or personnel or staff* or worker*))).ab. (0)

9 (acceptab* adj (surgeon* or doctor* or primary care provider* or general practitioner* or GP* or physician* or specialist* or nurse* or therapist*)).ab. (1)

10 or/4-9 (543)

11 (acceptab* adj4 (surgical* or surgery or surgeries or operat* or procedur* or treat* or technique* or intervention* or trial* or excis* or resect* or transplant* or graft*)).ab. (840)

12 (acceptab* adj4 (perisurg* or peri-surg* or perioperat* or peri-operat* or intraop* or intra-op* or midoperat* or mid-operat* or midsurg* or mid-surg*)).ab. (23)

13 (acceptab* adj4 (patient* or inpatient* or outpatient* or person* or people or adult* or m?n or wom?n or p?ediatric* or child* or baby or babies or infant* or juvenil* or adoles* or youth*)).ab. (547)

14 (acceptab* adj4 ((healthcare or medical) adj (professional* or personnel or staff* or worker*))).ab. (8)

15 (acceptab* adj4 (surgeon* or doctor* or primary care provider* or general practitioner* or GP* or physician* or specialist* or nurse* or therapist*)).ab. (27)

16 acceptab*.ab. /freq=2 (1070)

17 or/11-15 (1310)

18 17 and 16 (463)

19 10 or 18 (844)

20 3 and 19 (79)

21 limit 20 to yr="2000 -Current" (79)

Key:

* = truncation

? = wildcard for 0-1 letters

ti,ab = terms in either title or abstract fields

adj3 = terms within three words of each other (any order)

/freq=2 = terms occur a frequency of 2 or more times

Cochrane Central Register of Controlled Trials (CENTRAL)

via Wiley http://onlinelibrary.wiley.com/

Date range: Issue 10 of 12, October 2023
Date searched: 9th November 2023

Records retrieved: 1895

#1 [mh "Surgical Procedures, Operative"[mj]] 62187

#2 [mh /su] 80293

#3 (surgical* or surgery or surgeries):ti,ab 250401

#4 {or #1-#3} 305849

#5 acceptab*:ti 3487

#6 (acceptab* NEAR/3 (surgical* or surgery or surgeries or operat* or procedur* or treat* or technique* or intervention* or trial* or excis* or resect* or transplant* or graft*)):ab 7246

#7 (acceptab* NEAR/3 (perisurg* or peri NEXT surg* or perioperat* or peri NEXT operat* or intraop* or intra NEXT op* or midoperat* or mid NEXT operat* or midsurg* or mid NEXT surg*)):ab 35

#8 (acceptab* NEAR/3 (patient* or inpatient* or outpatient* or person* or people or adult* or m?n or wom?n or p?ediatric* or child* or baby or babies or infant* or juvenil* or adoles* or youth*)):ab 4982

#9 (acceptab* NEAR/3 ((healthcare or medical) NEXT (professional* or personnel or staff* or worker*))):ab 26

#10 (acceptab* NEAR/3 (surgeon* or doctor* or primary care provider* or general practitioner* or GP* or physician* or specialist* or nurse* or therapist*)):ab 1240

#11 {or #5-#10} 14568

#12 #4 and #11 with Publication Year from 2000 to 2023, in Trials 1895

Key:

MeSH descriptor = indexing term (MeSH)

[mj] = focussed subject heading

/su = surgery as a floating subheading

* = truncation

? = wildcard for 0-1 letters

ti,ab = terms in either title or abstract fields

near/3 = terms within three words of each other (any order)

next = terms are next to each other.

International HTA database

via https://database.inahta.org/

Date range: Inception – 9th November 2023
Date searched: 9th November 2023

Records retrieved: 72

All ((surgical* OR surgery OR surgeries) AND acceptab*)

Publication Year 2000 - 2023

= 72

Key:

* = truncation

ClinicalTrials.gov

via https://clinicaltrials.gov/

Date searched: 9th November 2023

Records retrieved: 109

2 separate searches were used, retrieving 109 records in total, which were imported into EndNote 21 and deduplicated.

Other Terms: ((acceptable or acceptability) AND (surgical OR surgery OR surgeries OR operation OR excision OR resection OR transplant OR graft)) = 105

Other Terms: ((acceptable or acceptability) AND (perisurgical OR perioperative OR intraoperative OR midsurgical OR midoperative)) = 4

WHO International Clinical Trials Registry Platform (ICTRP)

via https://trialsearch.who.int/

Date searched: 9th November 2023

Records Retrieved: 43

2 separate searches were used, retrieving 43 records in total, which were imported into EndNote 21 and deduplicated.

Title: (acceptable OR acceptability)

Intervention: (surgical OR surgery OR surgeries OR operation OR excision OR resection OR transplant OR graft) = 41

Recruitment Status: ALL

Title: (acceptable OR acceptability)

Intervention: (perisurgical OR perioperative OR intraoperative OR midsurgical OR midoperative) = 2

Recruitment Status: ALL
